# A New Species of Eucnemidae (Coleoptera: Elateroidea) with Its Complete Mitogenome and Mitogenome-Based Phylogenetic Analysis

**DOI:** 10.3390/biology15171453

**Published:** 2026-08-25

**Authors:** Ziye Meng, Jyrki Muona, Yongying Ruan, Qijiao Chen, Xiaoqin Chen, Qingfu Chen

**Affiliations:** 1 Buckwheat Industry Technology Research Center, School of Life Sciences, Guizhou Normal University, Guiyang 550025, China; qijiaochen@126.com; 2 Museum of Natural History, University of Helsinki, 00014 Helsinki, Finland; jyrki.muona@helsinki.fi; 3 Plant Protection Research Center, Shenzhen Polytechnic University, Shenzhen 518055, China; yongyingruan@hotmail.com (Y.R.); chenxiaoqin@szpu.edu.cn (X.C.)

**Keywords:** *Microrhagus*, new species, mitochondrial genome, phylogenetic

## Abstract

We describe *Microrhagus ziwulingensis* Muona & Meng, sp. nov., from China. The genus *Microrhagus* Dejean, 1833, was previously represented in China by only two species. We sequenced and assembled the complete mitogenome of *M. ziwulingensis* (GenBank accession OK143440), which encoded 13 protein-coding genes (PCGs), 2 ribosomal RNA genes (rRNAs), 22 transfer RNA genes (tRNAs), and a putative control region with a total length of 15,843 bp. Overall, 36 species of Elateroidea were collected as the ingroup (for six of these species, two sequences of the same species submitted by different submitters were used). Eight species of Buprestoidea served as the outgroup. We constructed phylogenetic trees using maximum likelihood (ML) and Bayesian inference (BI) methods based on 13 protein-coding genes (PCGs) from mitochondrial genomes. The phylogenetic trees showed that all families within the superfamily Elateroidea, which was used as the ingroup, formed monophyletic groups. The topology differed from previous studies, showing that Rhagophthalmidae and Lampyridae formed a sister clade, and that Phengodidae + Lycidae, with Cantharidae, formed a sister clade. These discrepancies should be attributed to the use of a single type of molecular marker and the intense shortage of available sampling. This also indicates that mitochondrial genomic research on Eucnemidae, and even on the superfamily Elateroidea, still needs further expansion.

## Simple Summary

Eucnemidae are considered important contributors to forest nutrient cycling, yet their fauna remains poorly established in China, and molecular phylogenetic studies of the family are lacking. Here, we describe a new species of *Microrhagus* Dejean, 1833, *Microrhagus ziwulingensis* Muona & Meng, sp. nov., from the Chinese fauna, and report its complete mitogenome. The circular molecule includes 13 protein-coding genes, two rRNA genes, 22 tRNA genes, and a putative control region. Established on 13 protein-coding genes (PCGs), phylogenetic trees were rebuilt using maximum likelihood and Bayesian inference methods. The consequent topologies were well supported, providing reliable molecular data for future phylogenetic studies of Eucnemidae.

## 1. Introduction

The false click beetles (Elateroidea: Eucnemidae) are a medium-sized family, including about 185 genera and 1700 described species worldwide [1]. A handful of studies have shown that eucnemids constitute a substantial part of tropical forest beetle diversity and can act as sufficient indicators of forest structural heterogeneity [2,3]. Despite their ecological significance, Eucnemidae have received little research attention in China, and their species variety and faunal composition remain poorly understood. Among the 12 genera of the tribe Dirhagini (Reitter, 1911), *Microrhagus* Dejean, 1833, is notable as one of the most recognized and widely distributed. The genus presently contains 156 described species worldwide, including 17 recorded from the Palaearctic region [4,5,6]. Nevertheless, *Microrhagus* displays remarkable morphological variety and heterogeneity and is widely regarded as a taxonomically complex group that warrants extensive revision [7]. According to this study, two species are known from China. Herein, we represent a new species, *Microrhagus ziwulingensis* Muona & Meng, sp. nov., thereby increasing the genus’s faunal annals in China.

Although molecular phylogenetic analyses within Elateroidea have been extensively conducted [8,9,10,11,12,13], those specifically focusing on Eucnemidae are lacking. Few studies on Eucnemidae have primarily focused on intrageneric or geographic population analyses [14,15]; no analysis has employed whole mitochondrial genome data to infer phylogenetic relationships within the family. This deficiency has not only hindered accurate modifications to its generic classification but also hindered further investigations into the interior phylogeny of Eucnemidae.

The insect mitochondrial genome is one of the most comprehensively studied invertebrate genomes. The mitochondrial genomes of Coleoptera are relatively small, simple in structure, and exactly maternally inherited. The gene order and composition are relatively conserved and substantially correspond to the ancestral arrangement in insects, except for occasional tRNA rearrangements; other rearrangements are rare. These features have made mitochondrial genomes widely useful for reconstructing phylogenetic relationships among coleopteran families, where they provide substantial resolving power [16,17,18,19,20,21,22,23,24]. In contrast, Eucnemidae remains almost unrepresented in mitochondrial genome studies; complete sequences available in public databases are extremely scarce, and no intrafamilial phylogenetic analysis based on whole mitochondrial genomes has yet been attempted.

In this study, we present the complete annotated mitogenome of the new species (GenBank accession OK143440). We also characterize the structural features, base composition, codon usage bias, and gene arrangement of the Eucnemidae mitochondrial genome, using previously published representative sequences from Elateroidea. Phylogenetic relationships within Elateroidea were reconstructed from 13 protein-coding genes (PCGs) to lay the foundation for future molecular systematic analyses of Eucnemidae.

## 2. Materials and Methods

### 2.1. Sample Collection

The specimens of *M. ziwulingensis* sp. nov. were caught in Mt. Ziwuling, Shaanxi Province, China. They are deposited in SZPU (Shenzhen Polytechnic University, Shenzhen, Guangdong, China) and GZNU (Guizhou Normal University, Guiyang, Guizhou, China) under the sampling number of SZEU-0101.

For the present study, all specimens were collected using flight interception traps (FITs) and malaise traps (MTs) in July to August 2019. They were preserved in absolute ethanol for subsequent molecular experiments. The holotype and paratypes were prepared as dry specimens. Observations of the habitus and diagnostic characteristics were performed using a Zeiss Discovery V20 and Nikon SMZ645 microscope. Digital images were taken with a Canon 800D and the Helicon Focus 7.6.1 system. After dissection, the genital segments were immersed in proteinase K solution (20 mg/mL) and incubated in a water bath at 55 °C for 1 h to remove the surrounding tissues. The dissected genitalia were photographed and then mounted on slides for preservation.

The classification system and morphological terminology follow the work of Muona [4,25].

### 2.2. Sequencing, Assembly and Annotation

Total genomic DNA was extracted using a modified cetyltrimethylammonium bromide (CTAB) method and applied to 500 bp paired-end library construction using the NEBNext Ultra DNA Library Prep Kit for Illumina sequencing (New England Biolabs, Ipswich, MA, USA). Sequencing was carried out on the Illumina NovaSeq 6000 platform (BIOZERON Co., Ltd., Shanghai, China). Genome reassembly was performed using SPAdes v3.14.1 [26]. All protein-coding genes (PCGs) were predicted by comparison with homologous sequences from reference mitogenomes and by identifying open reading frames (ORFs) using the invertebrate mitochondrial genetic code. An annotation of 22 transfer RNA (tRNA) genes was performed using the MITOS2 WebServer “https://usegalaxy.org (accessed on 12 December 2025)” [27]. The circular mitochondrial genome map was visualized using the Map to Reference function in Geneious Prime 2025.1.2 “https://www.geneious.com” (accessed on 22 August 2025), with the mitogenome of *Stenothemus fukienensis* Wittmer, 1974, used as a reference.

### 2.3. Sequence Analyses

Currently, only three mitochondrial genomes of Eucnemidae are available in GenBank, namely *Melasis buprestoides* (KX087315), *Eucnemidae* sp. (MH923241), and *Eucnemidae* sp. (JX412858). Among them, *Eucnemidae* sp. (JX412858) represents only a partial mitogenome lacking protein-coding regions and was therefore excluded from subsequent analyses.

The complete mitogenome sequence of *M. ziwulingensis* sp. nov. was deposited in GenBank and assigned the accession number OK143440. The nucleotide composition analysis and comparison with the other two eucnemid mitogenomes, as well as the calculation of relative synonymous codon usage (RSCU) values, were performed using PhyloSuite v1.2.3 [28]. Nucleotide diversity (Pi value) of 13 PCGs among three eucnemid mitogenomes was estimated using DnaSP v6.12.03 [29] with a sliding-window analysis (window size 200 bp and step size 20 bp).

A total of 36 Elateroidea species were included in the ingroup for phylogenetic analysis (including the sequence submitted in this study). Among them, six species, namely *Ludioschema sulcicolle*, *Melanotus cribricollis*, *Rhagophthalmus lufengensis*, *Luciola kagiana*, *Cephalomalthinus imparicornis*, and *Prothemus sanguinosus*, were represented by two sequences deposited by different submitters. Therefore, the ingroup dataset consisted of 42 Elateroidea mitogenome sequences. Eight Buprestoidea taxa were designated as the outgroup. The species discussed in this study and their corresponding GenBank accession numbers are provided in Table 1.

Sequence alignment was conducted using the MAFFT v7.505 [30] module in the PhyloSuite package, with the G-INS-i strategy selected. The alignment type was nucleotide, with a maximum of 16 iterative refinements, a gap penalty of −1.53, and iterative refinement based on global pairwise alignment information. The resulting alignment was later optimized using MACSE v5.3 [31], which uses the Invertebrate Mitochondrial Genetic Code (genetic code 5), an SE_B_8 amino acid alphabet, and the BLOSUM62 scoring matrix.

### 2.4. Phylogenetic Analyses

Phylogenetic analyses were conducted using both maximum likelihood (ML) and Bayesian inference (BI) approaches. The ML model was selected using the ModelFinder v2.2.0 [32] module in PhyloSuite, with the sequence type set to DNA, using Invertebrate Mitochondrial Genetic Code. The Bayesian Information Criterion (BIC) was used for model selection, and the model was fitted to IQtree for phylogenetic inference. Phylogenetic tree construction was performed using IQtree 3 [33], employing the GTR + F (empirical base frequencies) + I (invariant sites) + R7 (FreeRate model with 7 categories) model. Bayesian analysis was performed with MrBayes 3.2.7 [34], using the GTR + I + Γ model with six substitution types (Nst = 6), invgamma rates, and four gamma categories. State frequencies were fixed to empirical values. Two independent MCMC runs, each with one cold and three heated chains, were performed for 2,000,000 generations, with sampling undertaken every 1000 generations. A 25% relative burn-in was applied. Convergence was confirmed by an average standard deviation of split frequencies (ASDSF) of 0.007501 (<0.01), a maximum PSRF of 1.006, and effective sample sizes (ESS) exceeding 500 for all parameters. A 50% majority-rule consensus tree was constructed, and clades with posterior probabilities (PP) ≥ 0.95 were considered strongly supported.

**Table 1:** Mitochondrial genomes used for phylogenetic analysis in the present study.

Superfamily	Family	Species	Accession Number	Reference
Elateroidea (Ingroup)	Eucnemidae	Eucnemidae sp.	MH923241	[16]
		*Melasis buprestoides*	KX087315	Direct Submission
		*Microrhagus ziwulingensis* sp. nov.	OK143440	present study
	Elateridae	*Cryptalaus yamato*	MK524933	Direct Submission
		*Cryptalaus larvatus*	NC_047286	[17]
		*Ludioschema sulcicolle*	MK792747	Direct Submission
		*Ludioschema sulcicolle*	NC_053929	[18]
		*Melanotus cribricollis*	MK792748	[35]
		*Melanotus cribricollis*	NC_053930	Direct Submission
		*Pyrophorus divergens*	NC_009964	[19]
		*Limonius californicus*	NC_028541	[20]
		*Agriotes hirayamai*	NC_069551	Direct Submission
		*Silesis erberi*	NC_085503	Direct Submission
		*Sinelater perroti*	OP613099	Direct Submission
		*Sternocampsus coriaceus*	OP613100	Direct Submission
	Rhagophthalmidae	*Rhagophthalmus lufengensis*	DQ888607	Direct Submission
		*Rhagophthalmus lufengensis*	NC_010969	[21]
		*Rhagophthalmus giganteus*	MK292104	[36]
		*Rhagophthalmus ohbai*	NC_010964	[21]
	Lampyridae	*Luciola kagiana*	OQ184181	Direct Submission
		*Luciola kagiana*	NC_072664	Direct Submission
		*Luciola parvula*	LC677171	Direct Submission
		*Nipponoluciola cruciata*	AB849456	Direct Submission
		*Aquatica lateralis*	LC677169	Direct Submission
		*Curtos fulvocapitalis*	NC_058281	[22]
		*Stenocladius bicoloripes*	MZ457899	[23]
	Phengodidae	*Brasilocerus* sp.	KJ938490	[37]
	Lycidae	*Platerodrilus* sp.	KU878647	[24]
		*Lycostomus* sp.	MN264644	[38]
		*Lycostomus* sp.	MT554396	Direct Submission
	Cantharidae	*Amphimorphus semifumatus*	OM021995	[39]
		*Cephalomalthinus imparicornis*	NC_086617	[40]
		*Cephalomalthinus imparicornis*	OQ221871	[40]
		*Cephalomalthinus guizhouensis*	OM021992	[39]
		*Cephalomalthinus laticollis*	OM021993	[39]
		*Lycocerus curvatus*	NC_086605	[40]
		*Prothemus sanguinosus*	NC_086618	[40]
		*Prothemus sanguinosus*	OQ221872	[40]
		*Stenothemus fukienensis*	NC_086611	[40]
		*Taiwanocantharis parasatoi*	NC_086612	[40]
		*Themus stigmaticus*	NC_086614	[40]
		*Themus luteipes*	NC_086615	[40]
Buprestoidea (Outgroup)	Buprestidae	*Agrilus sichuanus*	NC_064324	[41]
		*Agrilus ornatus*	NC_064400	Direct Submission
		*Coraebus diminutus*	NC_064326	[41]
		*Meliboeus sinae*	NC_064327	[41]
		*Sambus kanssuensis*	NC_080317	Direct Submission
	Callirhipidae	*Simianus niponicus*	KX035160	Direct Submission
	Elmidae	*Cuspidevia jaechi*	PQ510303	[42]
		*Stenelmis punctulata*	PQ510305	[42]

## 3. Results

### 3.1. Taxonomy

**Figure 1:**
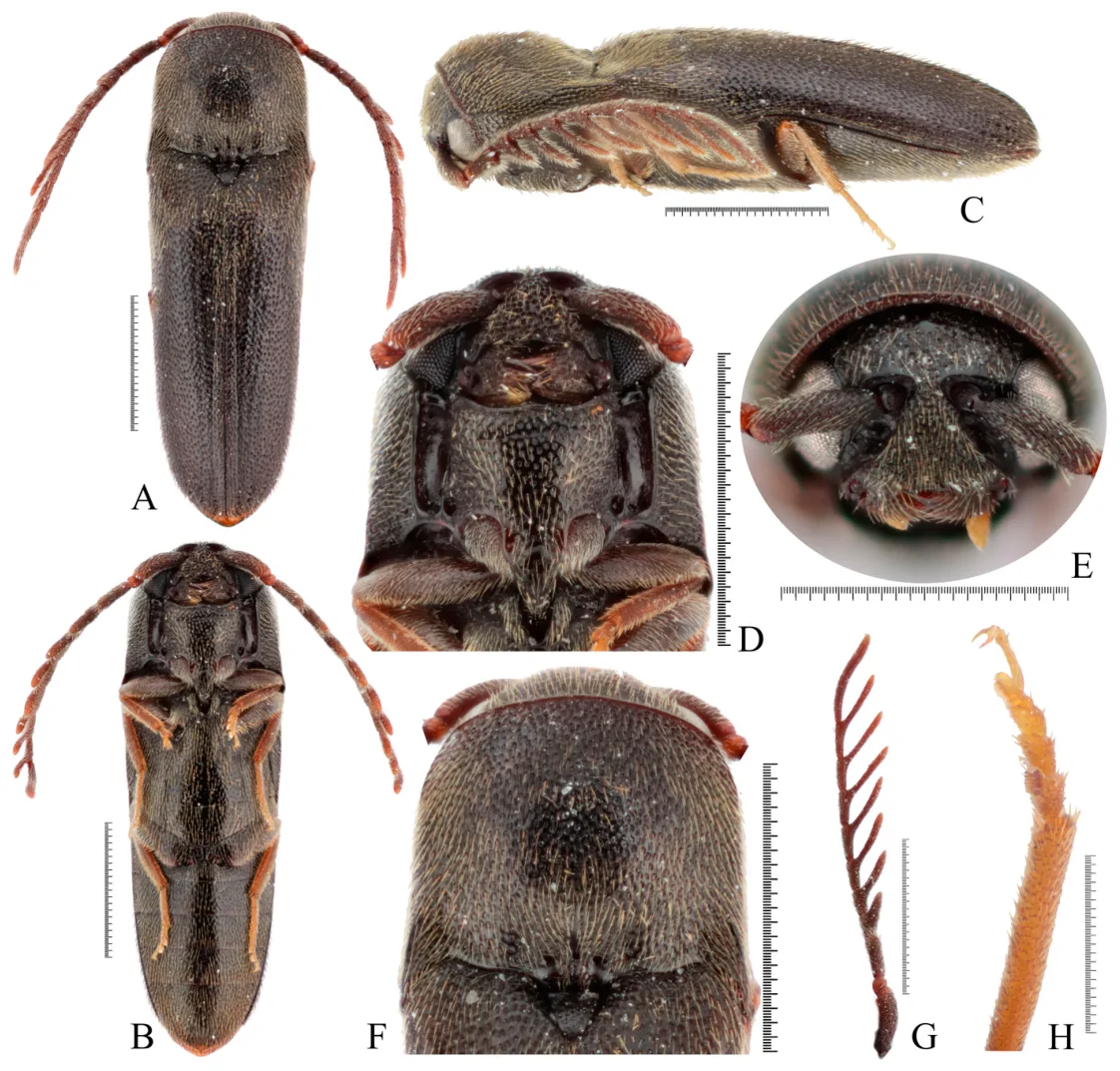
*Microrhagus ziwulingensis* sp. nov., holotype, male: (**A**) habitus, dorsal view, (**B**) ventral view, and (**C**) lateral view; (**D**) head, frontal view; (**E**) head and prosternum, ventral view; (**F**) pronotum; (**G**) antennae; (**H**) protarsus. Scale bar = 1 mm.

**Figure 2:**
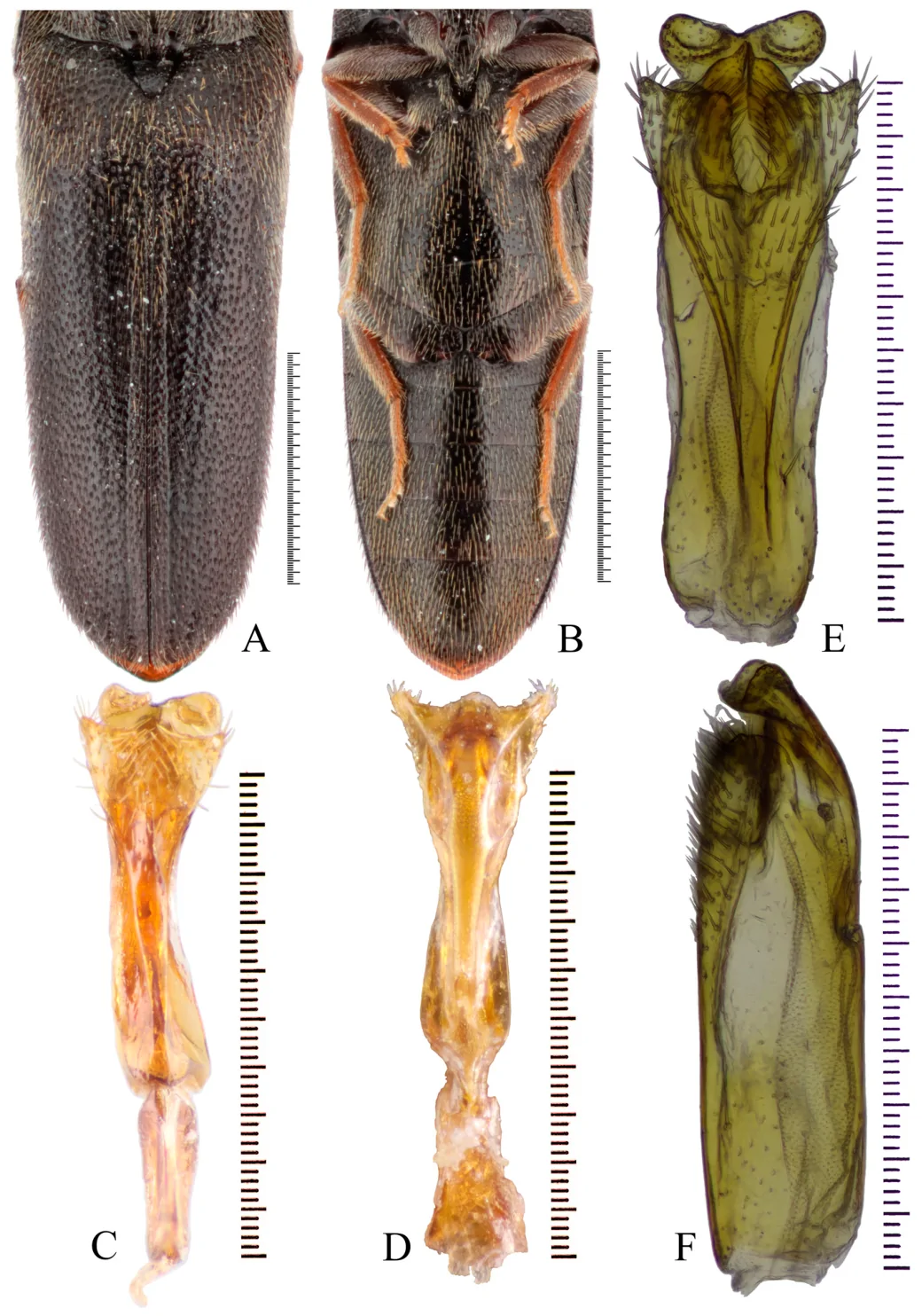
Characteristics of *Microrhagus ziwulingensis* sp. nov. (**A**) Elytra, (**B**) legs and abdomen, and (**C**) genitalia of holotype, male, ventral view; (**D**) genitalia of holotype, male, dorsal view; (**E**) genitalia of paratype, male, ventral view, basal plate absent; (**F**) genitalia of paratype, male, lateral view, basal plate absent (**A**,**B**) scale bar = 1 mm (**C**–**E**) scale bar = 0.5 mm.

**Figure 3:**
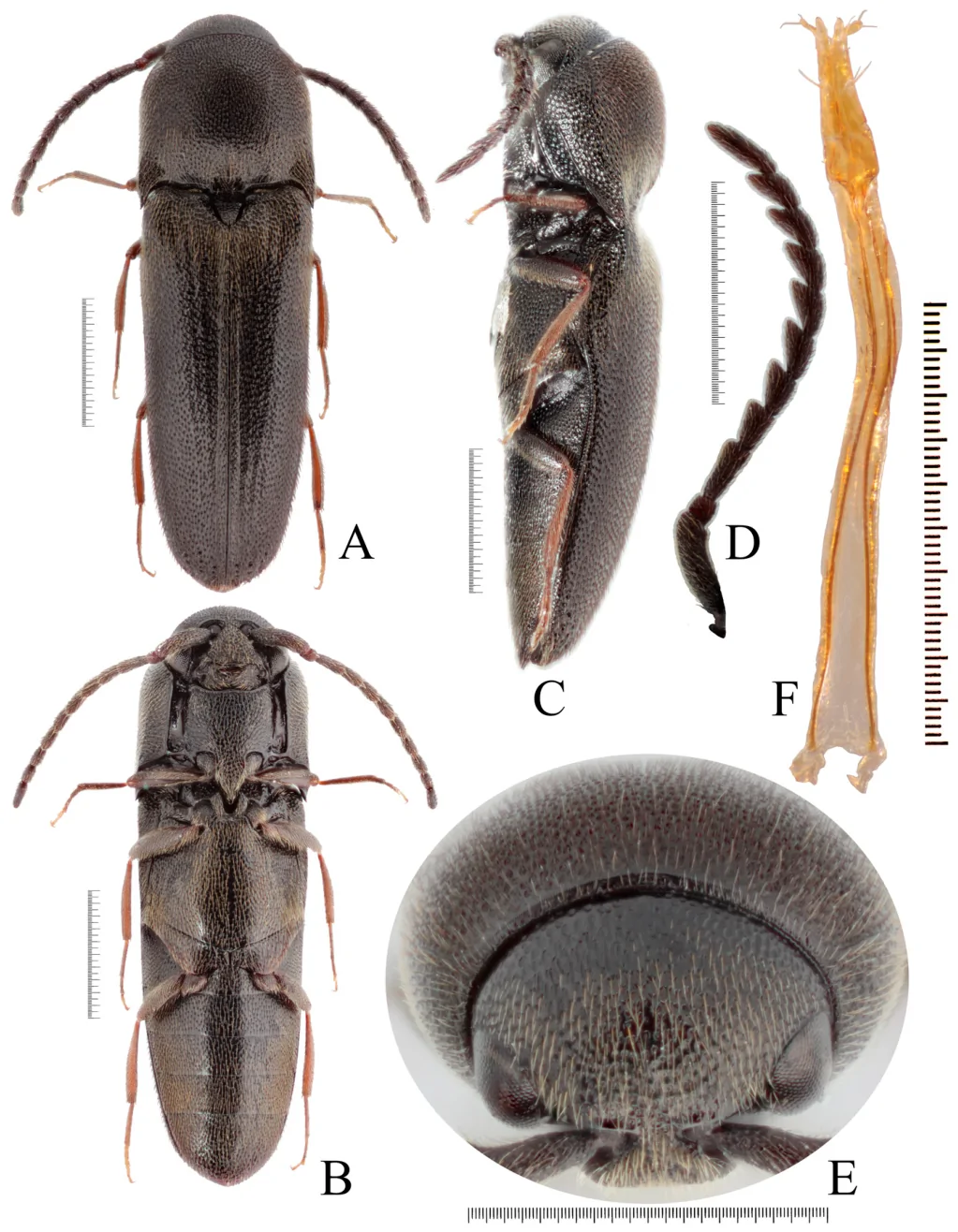
*Microrhagus ziwulingensis* sp. nov., paratype, female: (**A**) habitus, dorsal view; (**B**) habitus, ventral view’ (**C**) habitus, lateral view’ (**D**) antennae; (**E**) head, frontal view; (**F**) ovipositor, dorsal view (assembled from two images). (**A**–**E**) Scale bar = 1 mm; (**F**) scale bar = 0.5 mm.

*Microrhagus ziwulingensis* Muona & Meng sp. nov. (Figure 1, Figure 2 and Figure 3).

Zoobank registration link: urn: lsid: zoobank.org:pub:236D6F35-503F-4A39-9714-C7716E2716D3.

**Type materials**: Holotype, male, China, Shaanxi, Shihuigou Valley, Ziwuling National Nature Reserve, Yan’an City, 1256 m, E: 108.65577, N: 35.82770, 27–30.VII.2019, Jian Shen, Changping Ding, and Rui Dang leg. (SZPU). Paratypes: 1 female, same data as holotype, 6.VII.2019–1.VIII.2019, Jian Shen, Changping Ding, and Rui Dang leg. (SZPU); 1 male and 1 female, Huashugou Valley, Ziwuling National Nature Reserve, Yan’an City, 1256 m, E: 108.71143, N: 35.89698, 6.VII.2019-13.VII.2019, Jian Shen, Changping Ding, and Rui Dang leg. (GZNU); 1 male, Chenjiahe River, Ziwuling National Nature Reserve, Yan’an City, 1275 m, E: 108.65978, N: 35.88809, 3.VII.2019–13.VII.2019, Jian Shen, Changping Ding, and Rui Dang leg. (GZNU). Notes: 27 specimens were used for mitochondrial genome sequencing.

**Distribution**: China, Yan’an City.

**Etymology**: The species name refers to the type locality—Mt. Ziwuling.

**The key to *Microrhagus* of China**.

1. Male, body length > 4 mm; pronotum with a distinct median carina, a pair of shallow impressions at median portion of disc ......................................................................... *M*. *savioi*
— Male, body length < 4 mm; pronotum without median carina, disc lacking shallow median impressions .................................................................................. *M. ziwulingensis* sp. nov.
2. Females, body length > 6 mm; lateral carina of hind angle extending to anterior margin ............................................................................................................................ *M. klapperichi*
— Females, body length < 6 mm; Lateral carina of hind angle not extending to anterior margin .................................................................................................... *M. ziwulingensis* sp. nov.

**Description** (holotype, male): Body fusiform, dark, shiny, and moderately arched; surface punctate, covered with yellowish pubescence. Mandibles, antennae, and legs reddish brown; tarsi yellowish brown. Body length 3.8 mm, width 1.2 mm (Figure 1A–C).

**Head** wider than long, deeply inserted into prothorax, integument densely punctate, punctation circular, confluent at clypeal region. Vertex with a median line. Clypeus widest at lateral apices, about 9× as wide as at base between antennal sockets; medially arcuate, bearing long setae; punctures increasingly coarse and dense towards margin, becoming confluent and wrinkled. Eyes medium-sized, round (Figure 1D). Mandibles stout, bidentate, densely irregular punctate, with long pubescence (Figure 1E).

**Antennae** 2.9 mm long, extending beyond two-thirds of body length. Scape cylindrical, robust, about 5× as long as pedicel; pedicel shortest. Flagellomere I about 1.5× as long as flagellomere II; flagellomere II with a subtriangular lobe medially; flagellomeres III–VIII each bearing a pronounced apical process, progressively elongated toward flagellomere VIII; terminal flagellomere elongate, curved, about 2.8× as long as the preceding segment (Figure 1G).

**Pronotum** 1.1 mm long, widest at base of posterior angles, about 1.3× as wide as long at midline; lateral margins gradually narrowing in anterior third, then parallel-sided to base of posterior angles. Disc convex, with a fine ridge on anterior margin extending posteriorly and connecting with lateral carinae of hind angles; three small pits present at base of pronotum in front of scutellum. Disc densely punctate, punctures separated by about 0.5–1.0× their diameter; pubescence long, appressed posteriorly. Posterior angles strong, subtriangular, acute, apices directed posteriorly (Figure 1F). Hypomeron densely and irregularly punctate, with hairs directed posterolaterally; antennal grooves well developed, smooth, open anteriorly. Prosternum with punctures relatively sparser than on disc; anterior edge of prosternum forming an arched ridge (Figure 1E). Prosternal process convex at base, evenly depressed behind procoxae, deeply inserted into mesosternal cavity, gradually narrowed, acute at apex. Two pits near prosternal fossae deeper and more pronounced in males than in females (Figure 1E and Figure 3B).

**Scutellum** triangular, slightly longer than wide, disc flat, punctation sparser at apex than at base; basal margin straight, apex blunt (Figure 1F).

**Elytra** length 2.7 mm, about 4.2× as long as wide and 2.5× as long as pronotum, widest at humeri; lateral margins, gradually tapering towards apex; completely covering abdominal apex. Elytral striae deeply impressed at base, distinct, with convex interstices; sutural stria complete; striae 2–4 evanescent at about midlength; remaining striae restricted to basal portion. Surface finely and densely punctate, interspaces about 1.0–3.0× puncture diameter; punctures becoming coarser and confluent on humeri and at apices (Figure 2A).

**Metanepisterna** about 4.0× as long as wide, with subparallel sides. Metaventrite and metanepisterna finely and moderately densely punctate. Metaventrite with punctures larger and closer together laterally, evanescent on narrow medial and apical regions; interspaces mostly 0.5–2.0× puncture diameter. Metacoxal plates abruptly expanded medially, evenly narrowed laterally; finely and moderately densely punctate, with interspaces about 0.5–2.0× puncture diameter (Figure 2B).

**Legs** slender, setose. Protibiae shorter than profemora; meso- and metatibiae subequal to corresponding femora in length. Protibiae each with one apical spur and minute spine-combs on lateral side; protarsomere I bearing an apical sex-comb (Figure 1H). Meso- and metatibiae each with two well-developed apical spurs and regular spine-combs on lateral sides. Protarsus about 3/5 as long as protibia; meso- and metatarsi slightly shorter than corresponding tibiae. Claws simple (Figure 2B).

**Abdomen** elongate-oval, with simple, dense punctation; interspaces 3.0–5.0× puncture diameter. Punctation denser towards base and apex, with punctures coarser and partially confluent at apex; densely covered with long setae directed posteriorly. Sternite IX semi-oval, setae slightly denser distally. Tergite IX semi-oval, margin with long setae, apex incised; tergite X small, triangular (Figure 2B).

**Aedeagus** elongate, about 3.6× as long as wide; median lobe fused with lateral lobes, gently curved ventrally. Lateral lobes enlarged apically, densely covered with elongate, fine setae. Phallobase strongly constricted, 2.95× as long as wide, approximately one-third the length of aedeagus (Figure 2C–F).

**Male paratypes**: Body length 3.7–3.9 mm, body width 1.1–1.3 mm, elytral length 2.6–2.7 mm, pronotal length 1.0–1.2 mm, antennal length 2.1–2.2 mm.

**Description (female paratype)**: Similar to male but shinier, with comparatively sparser pubescence. Pronotum more convex posteromedially, anterior margin more rounded. Antennae and legs darker than those of male. Measurements: body length 5.0 mm, body width 1.7 mm, elytral length 3.5 mm, pronotal length 1.3 mm, antennal length 2.8 mm (Figure 3A–E). Ovipositor extremely slender, apically cleft medially, with long setae on both sides (Figure 3F).

**Natural history**: The species occurred at elevations of about 1200 m in the Ziwuling National Nature Reserve (Figure S1). At the collecting site, where FITs and MTs collected samples, the dominant trees were oak, white birch, aspen, and Chinese pine. The vegetation pattern is characteristic of a temperate deciduous broad-leaved forest, representing a natural secondary forest. The collecting period was concentrated from early July to early August. In contrast, in other regions (e.g., Guangdong, Hunan, Yunnan, etc.), adults are usually found in May and June. The difference in adult activity period may be related to temperature, but this requires further observation and data from a broader geographic range, as additional influencing factors may be involved.

### 3.2. Nucleotide Composition

The full circular mitogenome of *M*. *ziwulingensis* sp. nov. was 15,843 bp in length. All of the typical 37 mitochondrial genes were found in the genome, including 13 protein-coding genes (PCGs), 22 transfer RNA genes (tRNAs), two rRNA genes (*rrnS* and *rrnL*), and one non-coding control region (D-loop). The GC content of the mt genome is 26.6%; the overall base composition was A (40.5%) > T (32.9%) > C (16.2%) > G (10.4%), with a strong A-T bias (73.4%). High A + T content is a well-documented universal feature of insect mitochondrial genomes. The transcription hypothesis of codon usage offers a possible explanation; the exact causes of this AT bias cannot be attributed to a single factor [43].

**Figure 4:**
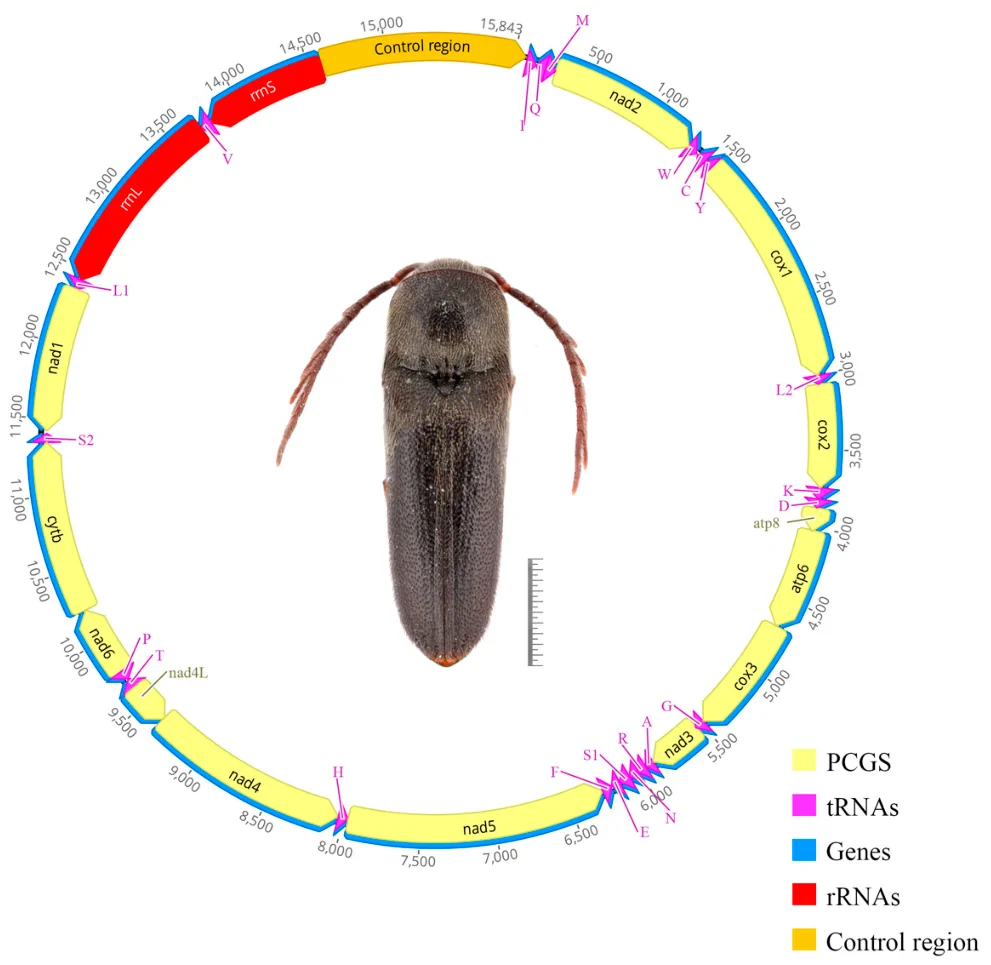
Gene map of *Microrhagus ziwulingensis* sp. nov. The inner circle shows the adult dorsal habitus (scale bar = 1 mm). The outer circle indicates the nucleotide positions (bp) in a clockwise direction. Protein-coding genes (PCGs) are shown in yellow, ribosomal RNA genes (rRNAs) in red, transfer RNA genes (tRNAs) in pink, and the control region in orange. The single-letter abbreviations for tRNA genes correspond to the following amino acids: A, tRNA-Ala (alanine); C, tRNA-Cys (cysteine); D, tRNA-Asp (aspartic acid); E, tRNA-Glu (glutamic acid); F, tRNA-Phe (phenylalanine); G, tRNA-Gly (glycine); H, tRNA-His (histidine); I, tRNA-Ile (isoleucine); K, tRNA-Lys (lysine); L1, tRNA-Leu (CUN) (leucine); L2, tRNA-Leu (UUR) (leucine); M, tRNA-Met (methionine); N, tRNA-Asn (asparagine); P, tRNA-Pro (proline); Q, tRNA-Gln (glutamine); R, tRNA-Arg (arginine); S1, tRNA-Ser (AGN) (serine); S2, tRNA-Ser (UCN) (serine); T, tRNA-Thr (threonine); V, tRNA-Val (valine); W, tRNA-Trp (tryptophan); Y, tRNA-Tyr (tyrosine).

The 13 concatenated protein-coding genes (PCGs) of *M. ziwulingensis* sp. nov. span 10,995 bp, representing 69.4% of the mitogenome. Overall, the PCG set exhibits negative AT-skew (−0.131) and GC-skew (−0.032) (Table S2). *atp8* is the smallest gene (153 bp), whereas *nad5* is the largest (1693 bp) (Table S1). At the third codon position, the CG content drops to 22.5%, significantly below the values observed at the first (32.4%) and second (32.4%) positions. Among the 13 PCGs, four (*nad1*, *nad4*, *nad4l*, and *nad5*) are encoded on the minority (N) strand. At the same time, the other nine are situated on the majority (J) strand (Figure 4, Table S1). Additionally, all PCGs initiate with the ATN start codon (ATA or ATG) and end with the putative terminal codons TAA or TAG (Table S1).

Neither the *M. ziwulingensis* sp. nov. we sequenced nor the previously reported eucnemid mitogenomes exhibited gene rearrangements, duplications, or deletions, indicating the absence of significant rearrangement events during their evolutionary divergence. This molecular stability is remarkable, as gene rearrangements have been documented in Lampyridae and Cantharidae [23,39]. It suggests that the mitochondrial genomes of the eucnemid species examined in this study have remained highly stable throughout evolution, and this conservatism provides a reliable source of sequence data for phylogenetic analyses.

**Figure 5:**
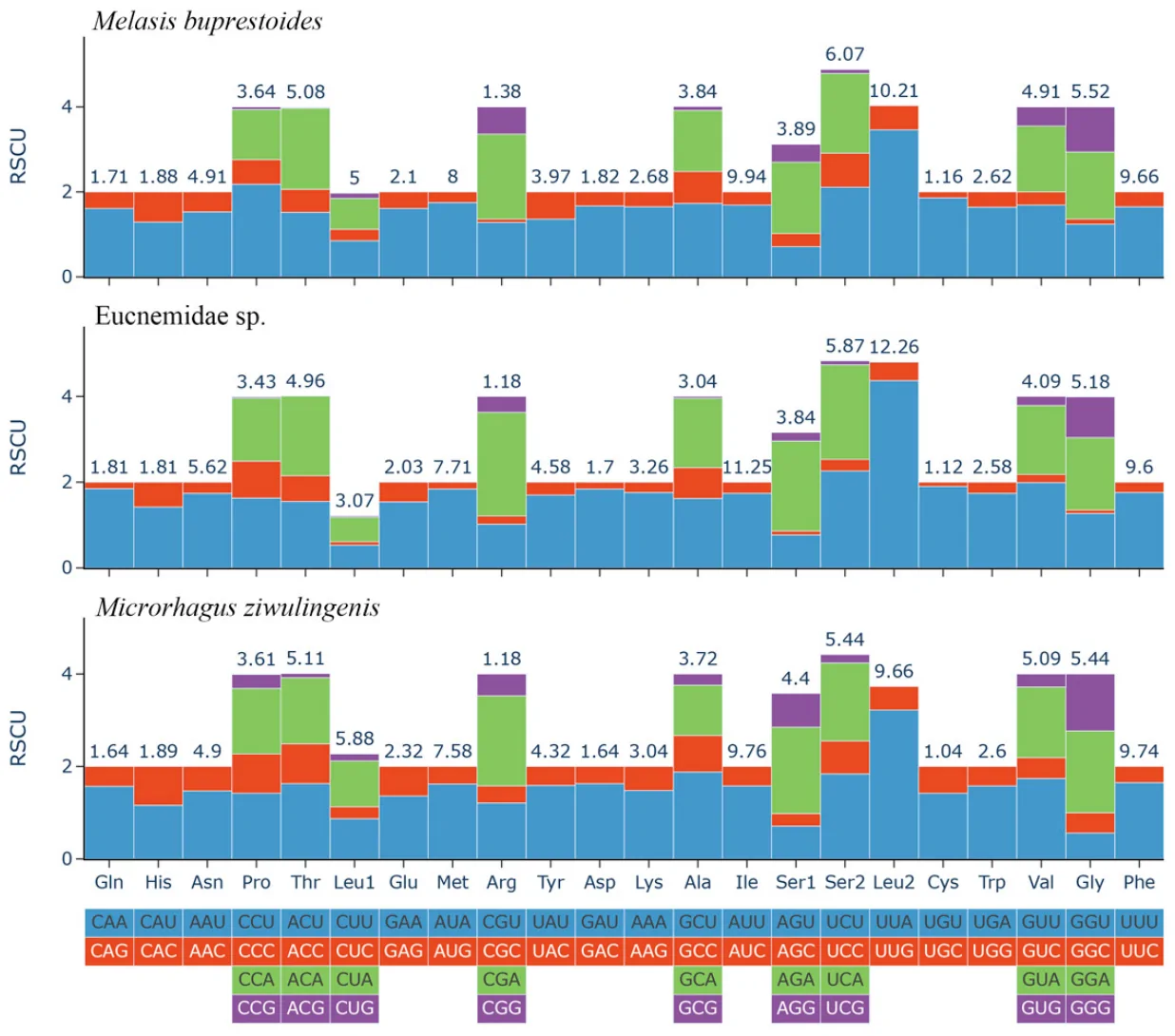
Relative synonymous codon usage (RSCU) of 13 protein-coding genes of *Microrhagus*.

The relative synonymous codon usage (RSCU) profiling of the three eucnemids mitogenomes revealed the most frequently used codon preferences: for *M. ziwulingensis* sp. nov, Leu2 (UUA), Arg (CGA), Ala (GCU), Ser1 (AGA), and Ser2 (UCU); for Eucnemidae sp., Leu2 (UUA), Arg (CGA), Ser2 (UCU), Ser2 (UCA), and Ser1 (AGA); and for Melasis buprestoides, Leu2 (UUA), Arg (CGA), Ser2 (UCU), Pro (CCU), and Ser2 (UCA). Furthermore, all codons with bars ≥ 1.5 exclusively end in A or U (UUU, AUU, UAU, AAU, AUA, UUA, etc.) (Figure 5). Regarding codon usage bias, partially similar results have been observed in studies of insects from the families Elateridae and Lampyridae [44,45]. The transcriptional hypothesis about codon usage bias is merely one of numerous hypotheses [46,47], and codon usage bias arises from the integrated action of multiple factors, among which mutational pressure is recognized as the primary initiator of its formation [48,49]. In addition to mutational pressure, natural selection serves as another major driving force. Codon usage bias analysis provides a means to determine which evolutionary force predominates in shaping codon usage patterns. Such as in Odonata, analyses of 31 mitochondrial genomes indicate that selection pressure is the primary evolutionary force driving codon usage bias [50]. Based on a sufficiently extensive dataset, codon usage bias analysis can be used to infer the primary drivers of codon usage bias in Eucnemidae.

In the *M*. *ziwulingensis* sp. nov. mitogenome, 14 gene overlaps ranging from 1 to 7 bp collectively span 32 bp, with the longest interval (7 bp) located at the *nad4*-*nad4l* junction; seven intergenic spacers of 1–73 bp add up to 107 bp, with the largest (73 bp) lying between the control region and *trnI*, and the second-largest (17 bp) located between *nad1* and *trnL1*. Consistent patterns are observed in two known mitogenomes: Eucnemidae sp. exhibits 14 overlaps (1–8 bp; 43 bp total) and 10 spacers (2–568 bp; 737 bp total), the longest spacer (568 bp) also being between control region and *trnI*, and the second-largest (45 bp) between *nad5* and *trnH*, whereas *M. buprestoides* harbors 19 overlaps (1–7 bp; 39 bp total) and 11 spacers (1–35 bp; 97 bp total), with the greatest intergenic distance (35 bp) situated between *trnY* and *cox1* (Table S1).

### 3.3. Transfer and Ribosomal RNA Genes

The positions of all 22 typical transfer RNA genes (tRNAs) were located in *M*. *ziwulingensis* sp. nov. (Table S1). Of these, 14 tRNAs are encoded on the majority (J) strand, while the remaining eight are encoded on the minority (N) strand. The total length of the 22 tRNAs in *M*. *ziwulingensis* sp. nov. was 1425 bp, accounting for 8.99% of the whole genome. The sizes of the 22 tRNAs range from 61 (*trnR*, *trnY*) to 71 bp (*trnK*). All 22 tRNAs indicated a negative AT-skew (−0.028) and a positive GC-skew (0.344) (Table S2).

The 22 tRNA genes all folded into the canonical cloverleaf secondary structure comprising four length-conserved arms: the ACC (aminoacyl acceptor arm), the dihydrouridine (DHU) arm, the anticodon stem that determines tRNA isotype, and the TΨC arm. The only deviation was observed in *trnS1*, which lacks the DHU arm—a condition that is ubiquitous across metazoans [51]. The anticodon loops of all tRNAs were highly conserved, each containing 7 bp, whereas the DHU and TΨC loops exhibited length heterogeneity (Figure S2). In addition to canonical AU and GC pairs, 21 GU wobble pairs were identified. A single unpaired nucleotide was also present within the aminoacyl stem.

The absence of the DHU arm in *trnS1* has been a highly recurrent observation in studies of mitochondrial genomes across numerous insect species [42,52,53]. In bilaterians, mitochondrial tRNA structures frequently depart from the standard cloverleaf model and may even be absent. Lang et al. characterized this process as a deteriorative evolutionary trajectory for mitochondrial tRNAs, in which canonical tRNA structural elements are modified or deleted, with compensatory mechanisms evolving accordingly [54].

Both rRNA genes (*rrnL* and *rrnS*) are encoded on the minority (N) strand, totaling 2078 bp. The large-subunit rRNA (*rrnL*) is interposed between *trnL1* and *trnV* and spans 1272 bp, whereas the small-subunit rRNA (*rrnS*) lies between *trnV* and the control region and comprises 806 bp (Table S1). Both genes exhibit a pronounced AT bias, reaching 81.5% (Table S2). Additionally, the rRNA region displays a negative AT-skew (−0.028) and a positive GC-skew (0.344) (Table S2).

In phylogenetic studies of Elateroidea, the *rrnL* fragment has been widely employed.

Previous systematic studies of Elateriformia by Kundrata & Bocak [9], Kundrata et al. [10], and Levkanicová & Bocák [55] provide valuable insights for phylogenetic analyses of Eucnemidae.

### 3.4. Nucleotide Diversity Analysis

**Figure 6:**
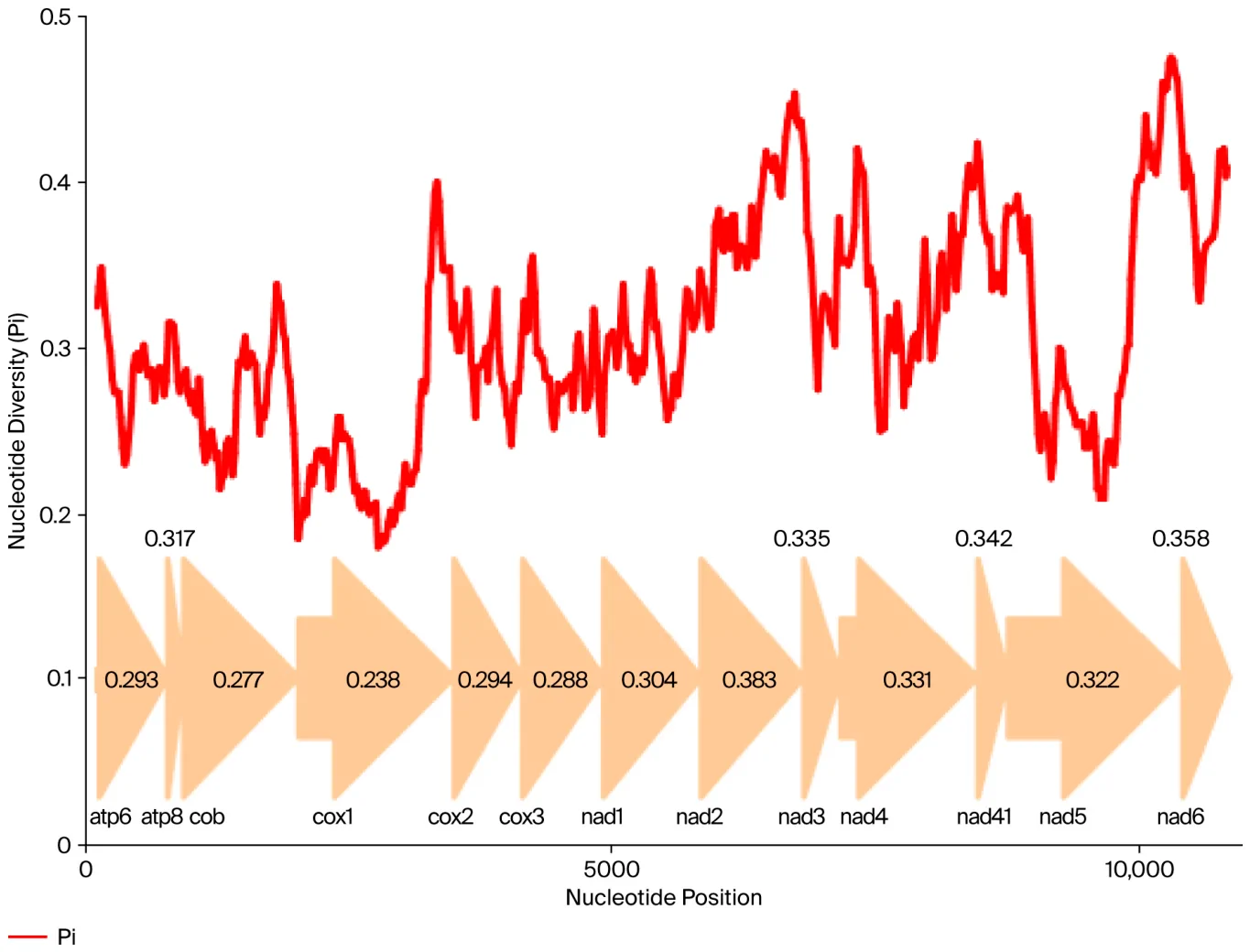
Sliding-window analysis across the 13 PCGs of three aligned Eucnemidae mitogenomes. The red curve indicates Pi values calculated using a sliding window approach. Orange arrows below represent the relative positions and transcriptional directions of the 13 protein-coding genes (atp6, atp8, cob, cox1, cox2, cox3, nad1, nad2, nad3, nad4, nad4l, nad5, and nad6). The numerical values beneath or above each gene indicate the corresponding nucleotide diversity (Pi).

The sliding window analysis concerning the nucleotide diversity (Pi values) of the 13 aligned PCGs among the three Eucnemidae mitogenomes, Eucnemidae sp., *M. buprestoides,* and *M*. *ziwulingensis* sp. nov., is shown in Figure 6. This demonstrates the high degree of nucleotide variation within different genes. Nucleotide diversity values range from 0.176 (*cox1*) to 0.348 (*atp8*) in these three species. In all PCGs, *atp8* (Pi = 0.348) shows the highest variability, followed by *nad6* (Pi = 0.308), *nad2* (Pi = 0.271), and *nad3* (Pi = 0.253), indicating comparatively high nucleotide diversity. The *nad1* (Pi = 0.200), *nad4l* (Pi = 0.200), *cox3* (Pi = 0.192), and *cox1* (Pi = 0.176) genes, with relatively low nucleotide diversity, indicate that they are relatively conserved in 13 PCGs. The extent of sequence variability across different gene fragments determines their potential utility as candidate markers for genetic investigations and taxonomic identification. In the case of *Krishna*, *cox1* was the least variable and slowest-evolving gene, whereas *nad4* and *nad2* exhibited the greatest variability and the fastest evolutionary rates. Consequently, these two genes are considered more suitable candidate markers for population genetic research and taxonomic identification within the genus *Krishna* [56]. This observation is consistent with earlier studies on Lamiinae, in which *atp8*, *nad6*, and *nad2* were likewise recommended as suitable markers for population genetics and taxonomic delimitation [57].

### 3.5. Phylogenetic Relationships

**Figure 7:**
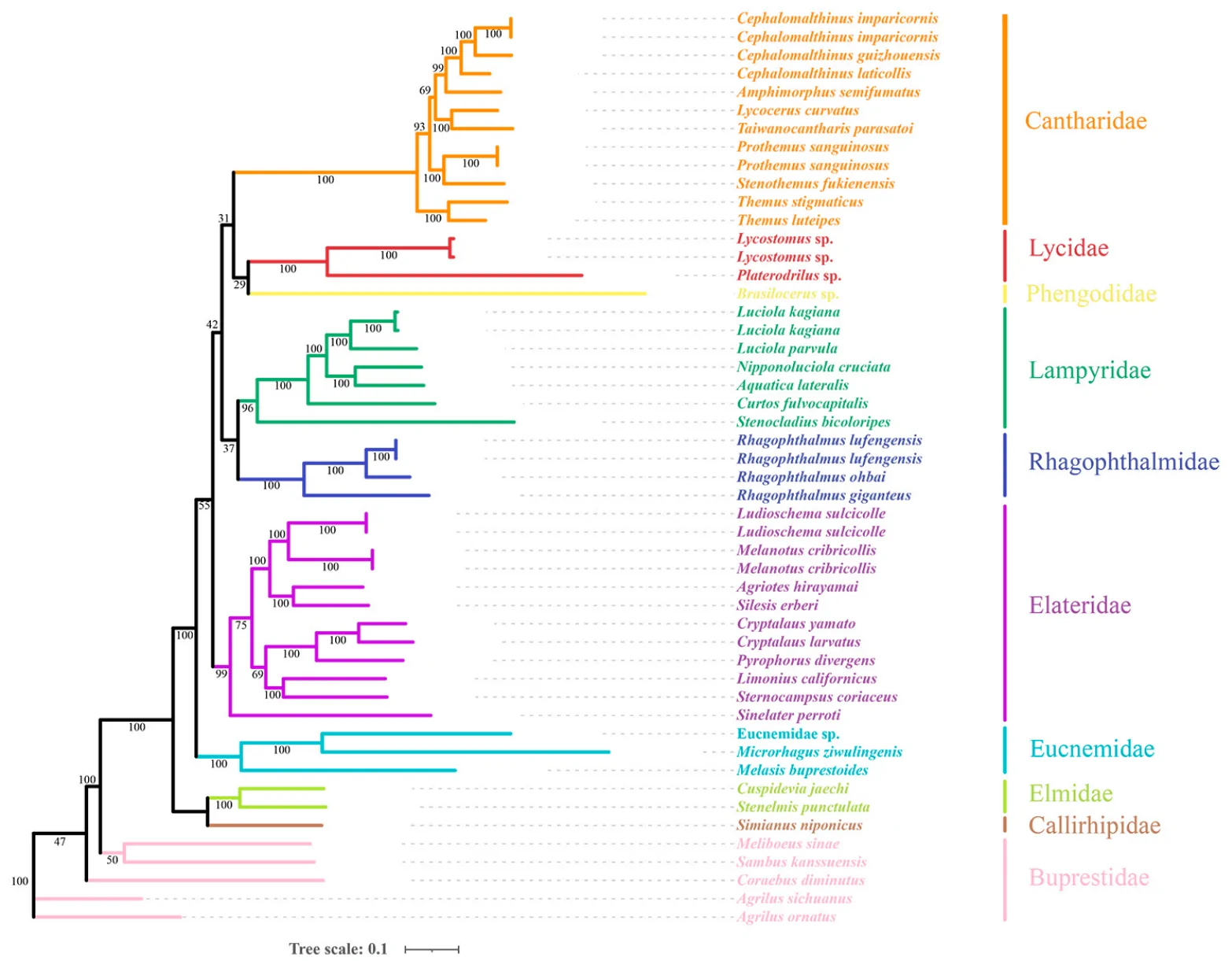
Phylogenetic tree of Elateroidea based on 13 PCGs from ML method.

Maximum likelihood (ML) and Bayesian inference (BI) analyses constructed practically similar tree topologies. All ingroup families were supported as monophyletic, and Elateroidea formed a large monophyletic group (Figure 7 and Figure S3). As noted by Kusy et al. and Muona & Taräväinen [13,58], when considering the extreme scenario that prioritizes monophyly alone, the entire Elateroidea could be treated as a single family. *M. ziwulingensis* sp. nov., grouped with the other two eucnemid species. Internal nodes within Elateroidea received moderate to high bootstrap support. The clustering of conspecific mitogenomes, including *Ludioschema sulcicolle*, *Melanotus cribricollis*, *Rhagophthalmus lufengensis*, *Luciola kagiana*, *Cephalomalthinus imparicornis*, and *Prothemus sanguinosus*, within single clades supported the stability of the topology.

Our results contrast with those of previous studies in the branching structure of the phylogenetic tree. In the studies by Bocakova et al., Kundrata & Bocak, and Kusy et al. [8,9,12], Phengodidae and Rhagophthalmidae were consistently recovered as a monophyletic group. Furthermore, in Kusy et al., Lampyridae, Phengodidae, Rhagophthalmidae, and Sinopyrophoridae together constituted the lampyroid clade [13]. In contrast, two sister-group relationships, Phengodidae + Lycidae and Rhagophthalmidae + Lampyridae, are reconstructed herein. Additionally, our phylogenetic tree does not reflect the previously established conclusion that Drilidae, Omalisidae, and Plastoceridae are placed within Elateridae, as these three families are absent from our dataset.

## 4. Discussion

The genus *Microrhagus* Dejean, 1833, is broadly distributed and comprises over 150 described species, characterized by morphological divergence and heterogeneity that have been considered to make its classification unstable. A comprehensive revision of the genus is thus needed. Before the study, only two species were reported from China. The new species described herein expands the Chinese fauna of this group, indicating that *Microrhagus* is more widely distributed in China than previously recognized. At present, 17 species are recorded from the Palaearctic region. As a major component of this zoogeographic realm, China likely maintains more undiscovered species of *Microrhagus*. This prospect emphasizes the need for specific faunistic surveys. The addition of species would provide taxonomic data to support the revision of the classification system.

The mitogenome of *M. ziwulingensis* sp. nov. in this study is highly conserved in terms of gene content, gene size, base composition, and codon usage. Among all protein-coding genes (PCGs), the *cox1* gene exhibited the lowest variation and the slowest evolutionary rate. In contrast, the *atp8*, *nad6*, *nad2*, and *nad3* genes may serve as potential molecular markers for distinguishing closely related species or for analyzing population genetic structure.

In our phylogenetic analyses of Elateroidea, we face a common challenge in mitogenome-based systematics, which is the limitation of taxon sampling. In this study, mitogenomic data were obtained for only three eucnemid species, representing two genera and one unnamed species. The limitations of sampling do not robustly support the relationships within the family Eucnemidae, making it difficult to draw complete and meaningful conclusions about its phylogeny. Furthermore, the absence of mitogenome data of families such as Drilidae makes it impossible to reproduce the previous phylogenetic hypotheses, thereby limiting the development of new suggestions regarding the existing classification. Consequently, the present study can only offer a tentative phylogenetic framework for Elateroidea.

An additional limitation arises from the use of mitochondrial sequences alone. The selection of genetic markers represents an essential factor that can influence the resulting topology in phylogenetic analyses. Mitogenomes are strictly maternally inherited and suffer from particular limitations compared with nuclear genes, such as pronounced heterogeneity. Therefore, the combined analysis of nuclear and mitochondrial genes is more effective for phylogenetic inference. Future studies should extend mitogenome sampling across Eucnemidae genera and contain additional genetic markers for combined analyses. Such works will support testing and refinement of the topologies reported herein, thereby providing a more robust phylogenetic framework.

## 5. Conclusions

A new species of the genus *Microrhagus* Dejean, 1833, *Microrhagus ziwulingensis* sp. nov., is described from the Ziwuling National Nature Reserve in Shaanxi Province, China, representing the third species of this genus recorded in China. The complete mitochondrial genome of *M. ziwulingensis* sp. nov. was sequenced and assembled. The circular mitogenome is 15,843 bp in length, falling within the typical range for insect mitogenomes, and comprises the standard set of 13 protein-coding genes (PCGs), 22 transfer RNA genes (tRNAs), two ribosomal RNA genes (*rrnL* and *rrnS*), and a control region. Structural and sequence characteristics, including genome size, gene composition, and nucleotide composition, are presented. The maximum likelihood (ML) and Bayesian inference (BI) trees, which were constructed based on the 13 PCGs, demonstrate that mitochondrial genomic data can serve as a fine tool for phylogenetic inference within Eucnemidae, recovering Eucnemidae as a well-supported monophyletic group and confirming the monophyly of all families within Elateroidea.

## Data Availability

The mitochondrial genome sequences analyzed in this study were downloaded from the GenBank database (https://www.ncbi.nlm.nih.gov/genbank/). Accession numbers are detailed in Table 1 and the main text. All original data and materials supporting the findings of this study are available within the article and its Supplementary Files. Requests for additional information should be addressed to the corresponding authors.

## Supplementary Materials

The following supporting information can be downloaded at: https://www.mdpi.com/article/10.3390/biology15171453/s1, Figure S1: locality of *M. ziwulingenis* sp. nov. in Ziwuling national nature reserve, Shaanxi, China.; Figure S2: Secondary structures of the 22 tRNAs of *Mi. ziwulingensis* sp. nov.; Figure S3: BI tree of Elateroidea based on 13 PCGs. Table S1: Mtiogenomic organization of three Eucnemidae species (*Eucnemidae* sp./*Melasis buprestoides*/*Microrhagus ziwulingenis* sp. nov.); Table S2: Nucleotide composition of mitogenomes among three Eucnemidae species (*Eucnemidae* sp./*Melasis buprestoides*/*Microrhagus ziwulingenis* nov.).

## Abbreviations

The following abbreviations are used in this manuscript:

SZPU	Shenzhen Polytechnic University
GZNU	Guizhou Normal University
FIT	flight interception traps
MT	Malaise traps
PCGs	Protein-encoding genes
RSCU	Relative synonymous codon usage
ML	Maximum likelihood
