# Supplementary material for: A New Species of Eucnemidae (Coleoptera: Elateroidea) with Its Complete Mitogenome and Mitogenome-Based Phylogenetic Analysis
Source: Biology (Basel). 2026 Aug 25;15(17):1453. doi: 10.3390/biology15171453 (PMC13564613; doi:10.3390/biology15171453)
Supplement: Supplementary file 1 [file biology-15-01453-s001.zip › biology-4402767-supplementary.pdf]

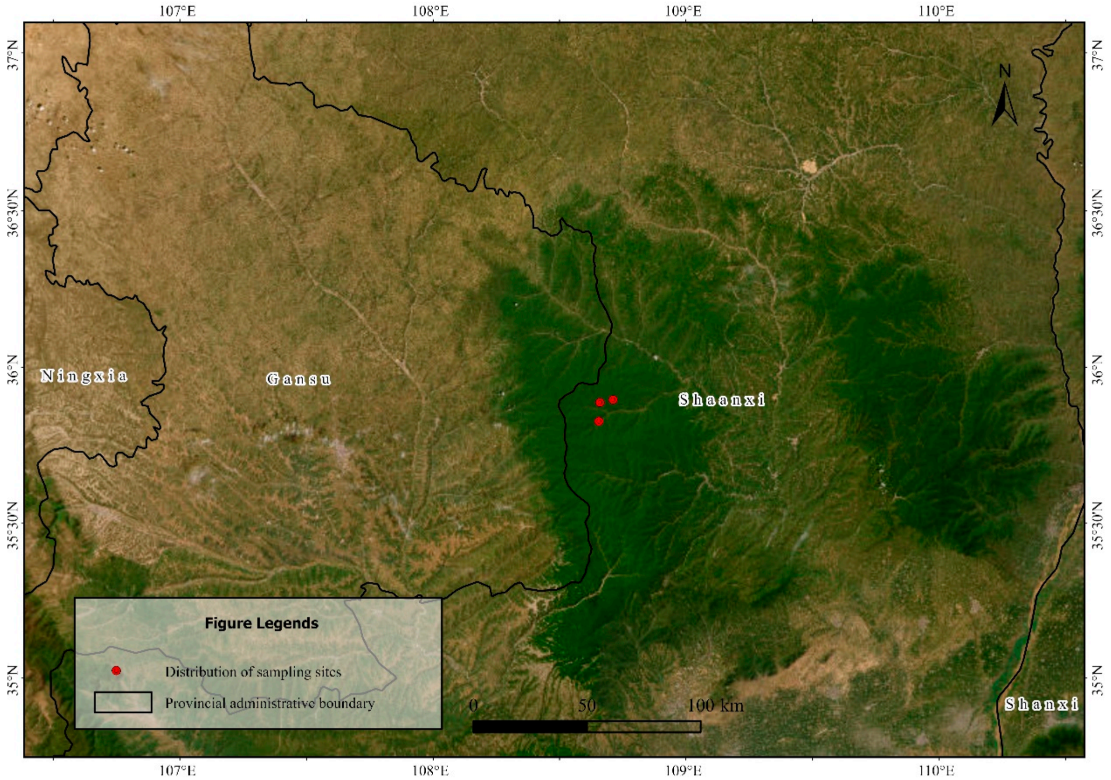

Figure S1. locality of *M. ziwulingenis* sp. nov. in Ziwuling National Nature Reserve, Shaanxi, China.

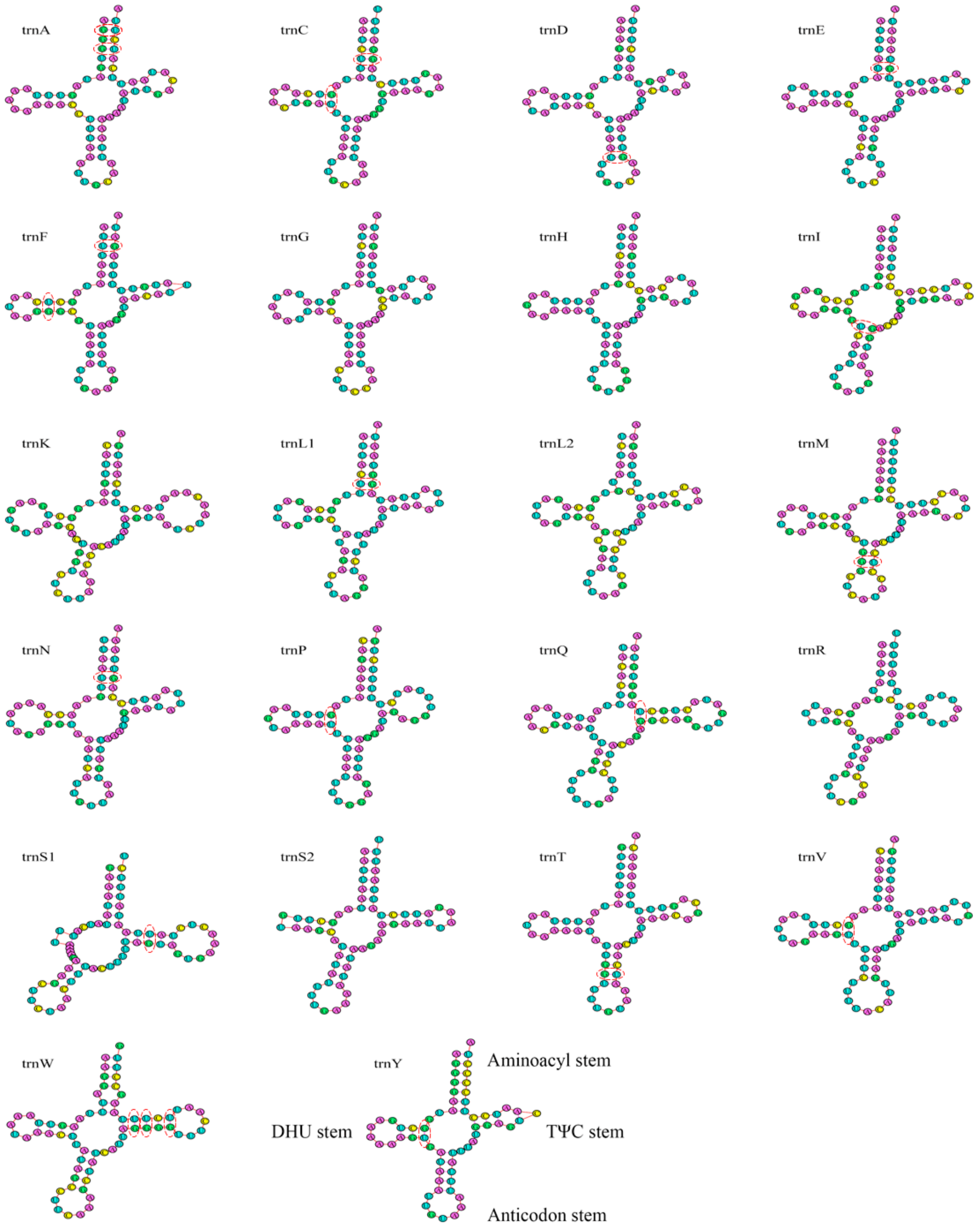

Figure S2. Secondary structures of the 22 tRNAs of *Mi. ziwulingensis* sp. nov.

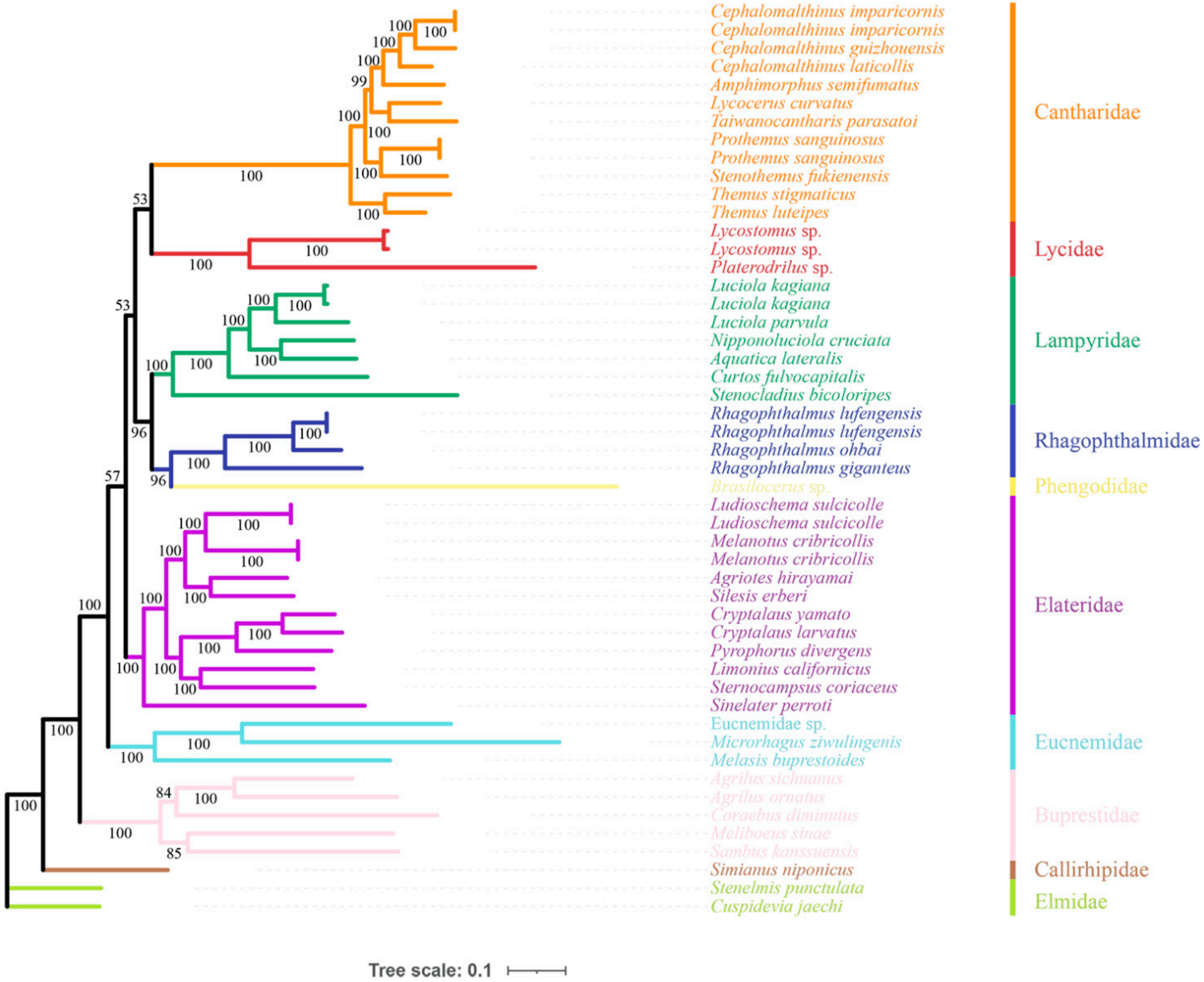

Figure S3. BI tree of Elateroidea based on 13 PCGs.

Table S1. Mtiogenomic organization of three Eucnemidae species (Eucnemidae sp./*Melasis buprestoides*/*Microrhagus ziwulingenis* sp. nov.).

Gene	Position		Size	Intergenic nucleotides	Codon		Strand	Identity
	From	To			Start	Stop		ES-MB/ES-MZ/MB-MZ/A
trnI	569/2/74	632/64/139	64/63/66	568/1/73			J/J/J	60.00/59.09/55.22/58.10
trnQ	630/62/137	698/130/205	69/69/69	-3/-3/-3			N/N/N	71.43/60.87/58.57/63.62
trnM	698/130/205	768/199/271	71/70/67	-1/-1/-1			J/J/J	86.11/80.28/68.57/78.32
nad2	787/207/272	1782/1199/1273	996/993/1002	18/7/--	ATA/ATT/ATA	TAA/TAA/TAA	J/J/J	63.63/54.44/60.75/59.60
trnW	1781/1202/1272	1845/1271/1340	65/70/69	-2/2/-2			J/J/J	83.10/73.91/73.24/76.75
trnC	1838/1298/1352	1899/1361/1414	62/64/63	-8/26/11			N/N/N	60.29/66.67/76.92/67.96
trnY	1900/1362/1415	1966/1423/1475	67/62/61				N/N/N	70.15/70.00/65.08/68.41
cox1	2001/1459/1477	3503/2961/3010	1503/1503/1534	34/35/1	ATT/ATT/ATG	TAA/TAA/T	J/J/J	75.38/74.66/74.53/74.86
trnL2	3499/2959/3011	3562/3020/3073	64/62/63	-5/-3/--			J/J/J	70.31/75.00/75.38/73.57
cox2	3581/3022/3074	4235/3688/3745	655/667/672	18/1/--	ATT/ATC/ATC	T/T/TAA	J/J/J	68.80/67.61/70.39/68.93
trnK	4236/3688/3746	4306/3754/3816	71/67/71	--/-1/--			J/J/J	69.44/79.17/69.44/72.69
trnD	4306/3754/3817	4368/3819/3878	63/66/62	-1/-1/--			J/J/J	80.60/76.19/85.07/80.62
atp8	4378/3821/3879	4524/3976/4031	147/156/153	9/1/--	ATA/ATT/ATC	TAA/TAA/TAA	J/J/J	58.75/63.12/68.35/63.41
atp 6	4518/3970/4028	5192/4629/4693	675/660/666	-7/-7/-4	ATG/ATG/ATA	TAA/TAA/TAA	J/J/J	70.96/67.11/71.00/69.69
cox 3	5192/4632/4693	5975/5415/5473	784/784/781	-1/2/-1	ATG/ATG/ATG	T/T/T	J/J/J	71.94/68.11/72.70/70.92
trnG	5976/5415/5474	6037/5476/5536	62/62/63	--/-1/--			J/J/J	82.54/74.60/84.38/80.51
nad 3	6047/5475/5534	6391/5828/5890	345/354/357	9/-2/-3	ATA/ATA/ATA	TAG/TAG/TAG	J/J/J	66.11/64.43/65.83/65.45
trnA	6390/5827/5889	6452/5888/5951	63/62/63	-2/-2/-2			J/J/J	87.50/77.27/79.69/81.49
trnR	6453/5889/5951	6517/5951/6011	65/63/61	--/--/-1			J/J/J	61.54/69.23/79.37/70.04
trnN	6518/5950/6011	6583/6013/6076	66/64/66	--/-2/-1			J/J/J	74.63/83.33/86.57/81.51
trnS1	6584/6015/6077	6650/6081/6143	67/67/67	--/1/--			J/J/J	80.88/82.09/85.29/82.76
trnE	6651/6082/6144	6714/6146/6208	64/65/65				J/J/J	86.36/83.08/83.33/84.26
trnF	6713/6145/6207	6776/6209/6269	64/65/63	-2/-2/-2			N/N/N	82.09/69.23/75.76/75.69
nad 5	6777/6210/6270	8439/7910/7962	1663/1701/1693		ATT/TTG/ATG	T/TAA/T	N/N/N	66.86/63.99/67.21/66.02
trnH	8485/7910/7964	8548/7975/8027	64/66/64	45/-1/1			N/N/N	61.19/61.54/65.67/62.80
nad4	8548/7976/8028	9873/9300/9345	1326/1325/1318	-1/--/--	ATG/ATG/ATG	TAG/TA/T	N/N/N	69.53/62.60/65.61/65.91

nad L	9867/9294/9339	10133/9584/9629	267/291/291	-7/-7/-7	ATC/ATG/ATG	TAA/TAA/TAA	N/N/N	59.45/59.11/67.70/62.08
trnT	10150/9585/9632	10212/9649/9695	63/65/64	16/--/2			J/J/J	73.85/75.00/73.85/74.23
trnP	10213/9650/9696	10276/9713/9759	64/64/64				N/N/N	71.21/83.08/81.54/78.61
nad 6	10279/9717/9762	10761/10184/10223	483/468/462	2/3/2	ATA/ATA/ATT	TAA/TAA/TAA	J/J/J	58.06/57.11/65.19/60.12
cob	10761/10184/10223	11891/11308/11344	1131/1125/1122	-1/-1/-1	ATG/ATG/ATG	TAG/TAA/TAA	J/J/J	71.78/69.85/73.32/71.65
trnS2	11890/11308/11344	11955/11372/11406	66/65/63	-2/-1/-1			J/J/J	69.70/68.18/80.60/72.83
nad1	11974/11391/11424	12900/12335/12371	927/945/948	18/18/17	TTG/ATA/ATA	TAA/TAG/TAG	N/N/N	68.03/65.51/70.04/67.86
trnL1	12901/12335/12369	12961/12396/12431	61/62/63	--/-1/-3			N/N/N	81.25/82.54/85.94/83.24
rrnL	12962/12396/12432	14214/13675/13703	1253/1280/1272	--/-1/--			N/N/N	71.68/71.31/76.53/73.17
trnV	14215/13675/13704	14288/13742/13771	74/68/68	--/-1/--			N/N/N	65.33/75.68/80.00/73.67
rrnS	14289/13742/13772	15042/14490/14577	754/749/806	--/-1/--			N/N/N	69.54/64.16/66.71/66.80

ES: Eucnemidae sp.; MB: Melasis buprestoides; MZ: Microrhagus ziwulingenis nov.; A: Average

Table S2. Nucleotide composition of mitogenomes among three Eucnemidae species (Eucnemidae sp./*Melasis buprestoides*/*Microrhagus ziwulingenis* nov.).

Regions	Size (bp)	T(U) (%)	C (%)	A (%)	G (%)	AT (%)	GC (%)	AT-skew	GC skew
Full genome	16170/15729/15843	35.2/32.7/32.9	13.4/16.1/16.2	43.1/41.6/40.5	8.4/9.6/10.4	78.3/74.3/73.4	21.8/25.7/26.6	0.101/0.119/0.103	-0.230/-0.254/-0.220
PCGs	10968/10899/10995	42.8/41.2/40.1	12.1/14.5/15	33.4/31/30.8	11.7/13.4/14.1	76.2/72.2/70.9	23.8/27.9/29.1	-0.123/-0.141/-0.131	-0.018/-0.039/-0.032
1st codon position	3656/3633/3665	36.2/33.9/33	11.3/13.6/14.2	36.5/34.4/34.7	16/18.1/18.2	72.7/68.3/67.7	27.3/31.7/32.4	0.005/0.008/-0.026	0.174/0.144/0.124
2nd codon position	3656/3633/3665	47.8/47.6/47.6	17.3/18.6/17.8	21/19.3/19.9	13.9/14.5/14.6	68.8/66.9/67.5	31.2/33.1/32.4	-0.389/-0.423/-0.409	-0.109/-0.122/-0.099
3rd codon position	3656/3633/3665	44.3/42/39.7	7.8/11.3/13	42.8/39.3/37.8	5.1/7.5/9.5	87.1/81.3/77.5	12.9/18.8/22.5	-0.018/-0.034/-0.025	-0.203/-0.199/-0.159
atp6	660/675/666	38.6/38.7/36.5	16.2/19.3/19.4	36.5/32.9/34.5	8.6/9.2/9.6	75.1/71.6/71.0	24.8/28.5/29.0	-0.028/-0.081/-0.027	-0.305/-0.354/-0.337
atp 8	156/147/153	40.4/38.8/37.9	10.9/17.0/17.0	44.9/39.5/40.5	3.8/4.8/4.6	85.3/78.3/78.4	14.7/21.8/21.6	0.053/0.009/0.033	-0.478/-0.562/-0.576
cox1	1503/1503/1534	36.5/35.0/34.1	16.9/18.5/19.9	31.9/31.5/30.2	14.7/15.0/15.8	68.4/66.5/64.3	31.6/33.5/35.7	-0.066/-0.052/-0.060	-0.069/-0.105/-0.115
cox2	667/655/672	36.6/36.6/33.3	15.0/17.1/18.2	37.3/36.6/36.9	11.1/9.6/11.6	73.9/73.2/70.2	26.1/26.7/29.8	0.010/0.000/0.051	-0.149/-0.280/-0.220
cox3	784/784/781	38.8/37.9/35.3	14.3/17.1/16.8	34.7/32.1/33.8	12.2/12.9/14.1	73.5/70.0/69.1	26.5/30.0/30.9	-0.056/-0.082/-0.022	-0.077/-0.140/-0.087
cob	1125/1131/1122	37.1/34.9/36.3	16.1/19.3/17.3	35.9/33.2/34.1	10.9/12.6/12.3	73.0/68.1/70.4	27.0/31.9/29.6	-0.016/-0.025/-0.030	-0.191/-0.211/-0.169
nad1	945/927/948	51.6/49.6/47.6	9.1/10.4/10.5	24.6/22.8/23.2	14.7/17.3/18.7	76.2/72.4/70.8	23.8/27.7/29.2	-0.356/-0.371/-0.344	0.236/0.250/0.278

nad2	993/996/1002	37.2/35.9/34.4	11.5/15.0/19.3	44.7/39.9/33.5	6.6/9.2/12.8	81.9/75.8/67.9	18.1/24.2/32.1	0.092/0.052/-0.013	-0.267/-0.237/-0.202
nad3	354/345/357	41.2/37.7/40.3	12.1/17.1/14.6	37.3/35.4/35.6	9.3/9.9/9.5	78.5/73.1/75.9	21.4/27.0/24.1	-0.050/-0.032/-0.063	-0.132/-0.269/-0.209
nad4	1325/1326/1318	51.6/48.8/47.3	7.8/9.3/10.6	27.2/24.7/25.4	13.4/17.2/16.6	78.8/73.5/72.7	21.2/26.5/27.2	-0.310/-0.327/-0.301	0.267/0.299/0.220
nad4L	291/267/291	56.4/53.2/51.5	3.8/7.5/8.9	26.1/22.8/25.1	13.7/16.5/14.4	82.5/76.0/76.6	17.5/24.0/23.3	-0.367/-0.399/-0.345	0.569/0.375/0.235
nad5	1701/1663/1693	48.8/48.2/46.3	7.9/9.3/10.2	30.1/26.9/27.0	13.2/15.6/16.5	78.9/75.1/73.3	21.1/24.9/26.7	-0.237/-0.283/-0.263	0.248/0.256/0.239
nad6	468/483/462	39.3/36.0/39.6	13.7/16.4/13.2	42.1/38.9/40.7	4.9/8.7/6.5	81.4/74.9/80.3	18.6/25.1/19.7	0.034/0.039/0.013	-0.471/-0.306/-0.341
rrnL	1280/1253/1272	47.9/45.4/47.7	5.4/6.9/5.0	34.8/33.1/34.6	11.9/14.6/12.7	82.7/78.5/82.3	17.3/21.5/17.7	-0.158/-0.157/-0.160	0.376/0.361/0.431
rrnS	749/754/806	45.0/43.9/44.9	6.3/6.5/6.2	36.0/34.1/35.5	12.7/15.5/13.4	81.0/78.0/80.4	19.0/22.0/19.6	-0.110/-0.126/-0.117	0.338/0.410/0.367
rRNAs	2029/2007/2078	46.8/44.8/46.6	5.7/6.7/5.5	35.3/33.5/34.9	12.2/14.9/12.9	82.1/78.3/81.5	17.9/21.6/18.4	-0.140/-0.145/-0.143	0.361/0.379/0.405
tRNAs	520/525/515	43.7/40.2/38.6	6.0/7.6/8.2	36.3/34.7/36.5	14.0/17.5/16.7	80.0/74.9/75.1	20.0/25.1/24.9	-0.091/-0.074/-0.028	0.404/0.394/0.344
